# Implementation of a consensus protocol for antibiotic use for bone and joint infection to reduce unnecessary outpatient parenteral antimicrobial therapy: A quality improvement initiative

**DOI:** 10.1017/ash.2021.250

**Published:** 2022-01-12

**Authors:** Hiroyuki Suzuki, Hilary J. Mosher, Brett H. Heintz, Daniel J. Livorsi

**Affiliations:** 1 Iowa City Veterans Affairs Health Care System, Iowa City, Iowa; 2 Department of Internal Medicine, University of Iowa Carver College of Medicine, Iowa City, Iowa; 3 Veterans Affairs Quality Scholarship Program

## Abstract

**Objective:**

We aimed to decrease the use of outpatient parenteral antimicrobial therapy (OPAT) for patients admitted for bone and joint infections (BJIs) by applying a consensus protocol to suggest oral antibiotics for BJI.

**Design:**

A quasi-experimental before-and-after study.

**Setting:**

Inpatient setting at a single medical center.

**Patients:**

All inpatients admitted with a BJI.

**Methods:**

We developed a consensus table of oral antibiotics for BJI among infectious diseases (ID) specialists. Using the consensus table, we implemented a protocol consisting of a weekly reminder e-mail and case-based discussion with the consulting ID physician. Outcomes of patients during the implementation period (November 1, 2020, to May 31, 2021) were compared with those during the preimplementation period (January 1, 2019, to October 31, 2020). Our primary outcome was the proportion of patients treated with OPAT. Secondary outcomes included length of hospital stay (LOS) and recurrence or death within 6 months.

**Results:**

In total, 77 patients during the preimplementation period and 22 patients during the implementation period were identified to have a BJI. During the preimplementation period, 70.1% of patients received OPAT, whereas only 31.8% of patients had OPAT during the implementation period (*P* = .003). The median LOS after final ID recommendation was significantly shorter during the implementation period (median 3 days versus 1 day; *P* < .001). We detected no significant difference in the 6-month rate of recurrence (24.7% vs 31.8%; *P* = .46) or mortality (9.1% vs 9.1%; *P* = 1.00).

**Conclusions:**

More patients admitted with BJIs were treated with oral antibiotics during the implementation phase of our quality improvement initiative.

Outpatient parenteral antimicrobial therapy (OPAT) was introduced in the 1970s and has been widely used for infectious diagnoses for which long-term intravenous (IV) therapy is needed. However, OPAT has been associated with higher costs, longer length of hospital stay (LOS), and lower patient satisfaction compared to oral therapy.^1,2^ Furthermore, OPAT puts patients at risk for catheter-related complications in addition to antibiotic-related complications.^3–6^ Up to ∼40% of OPAT is potentially unnecessary.^2^

Bone and joint infections (BJIs), such as osteomyelitis and septic arthritis, have been considered strong indications for OPAT. Although BJIs have traditionally been treated with at least 6 weeks of intravenous therapy, a small randomized control trial and several observational studies have suggested that oral antibiotic combination therapy might be as effective as intravenous (IV) therapy.^7–9^ In 2019, a randomized control trial (Oral versus Intravenous Antibiotics for Bone and Joint Infection, the OVIVA trial) reported that oral antibiotic therapy was not inferior to IV therapy for BJI.^10^ Following the OVIVA trial, a study investigated the economic impact of implementing the concept of the OVIVA trial for patients with BJI treated using OPAT. These researchers found that ∼80% of patients treated by OPAT were eligible for oral antimicrobial therapy and that changing to oral therapy would lead to a reduction of ∼20 days of intravenous therapy and £1,234 ($1,631) cost reduction per patient.^11^ Another study from an orthopedic hospital reported that, following implementation of the OVIVA trial protocol, two-thirds of patients previously treated with IV antibiotics were treated with oral therapy.^12^ They observed no difference in clinical outcomes as well as decreased LOS, reduced cost of antibiotic treatment, and increased drug-related complications, mainly gastrointestinal intolerance. Although those studies suggested that oral antibiotic therapy for BJI can be acceptable to physicians and has benefits, they were conducted in United Kingdom. Little evidence about implementing the OVIVA trial findings is available in the United States where antimicrobial resistance patterns and the availability of OPAT are different. How those studies affected the clinical practice of ID physicians in the United States remains unclear.

The Iowa City Veterans Affairs Health Care System (ICVAHCS) has an antimicrobial stewardship program with ID providers and an ID pharmacist overseeing all patients on inpatient antibiotic therapy through daily prospective audit and feedback. In this quality improvement initiative led by the antimicrobial stewardship program, we developed a consensus table among ID providers and ID pharmacists and applied the protocol to suggest oral antibiotics for BJI using the consensus table. We aimed to decrease the use of OPAT for patients admitted for BJI by applying the protocol.

## Methods

### Population and setting or context

We conducted a quality improvement initiative at ICVAHCS as a part of the Veterans Affairs Quality Scholarship program. Our population of interest included all inpatients admitted with BJI (native vertebral osteomyelitis, peripheral osteomyelitis, native joint septic arthritis, prosthetic joint infection and orthopedic hardware-related infection) for whom the ID service recommended treatment with at least 4–6 weeks of either IV or oral antibiotics. We did not include patients who had complete resection of the infected lesion (ie, amputation) because antibiotics were stopped after a short duration. We also excluded patients if the ID service recommended a short duration of therapy for skin and soft-tissue infection, even with evidence of chronic osteomyelitis. Although we limited our study to patients who had inpatient ID consultation, we believe we covered almost all patients with those conditions because ID consultations are almost always made for patients who may require OPAT and because the antimicrobial stewardship team recommends ID consultation if they have not been consulted already.

### Intervention

We began by developing a consensus table of oral antibiotics for BJI. First, based on available literature, we created a draft table for possible oral antibiotic regimens for BJI caused by specific organisms: *Staphylococcus aureus*, coagulase-negative *Staphylococcus*, *Streptococcus* spp*, Enterococcus* spp, enteric gram-negative rod organisms, *Pseudomonas aeruginosa*, or unknown organisms.^7,10,13–18^ Next, a survey questionnaire was distributed using REDCap among ID physicians and ID pharmacists at the University of Iowa Hospitals and Clinics (UIHC) and ICVAHCS. The questionnaire asked how frequently the provider recommends the specific oral antibiotic (1 “almost never” to 5 “very frequently”) in the setting where there is no contraindication for choosing oral antibiotics and the patient does not have a condition for which IV therapy is preferred, such as an undrained abscess or epidural abscess. According to the average score, antibiotics were classified as first-line or second-line oral options, or they were excluded from the table. In the first round of the survey, 17 or 29 survey recipients responded. In the second round of the survey, 11 or 29 survey recipients responded. After 2 rounds of the survey, we developed a consensus table, which we used for the protocol (Supplementary Fig. 1).

**Fig. 1.**
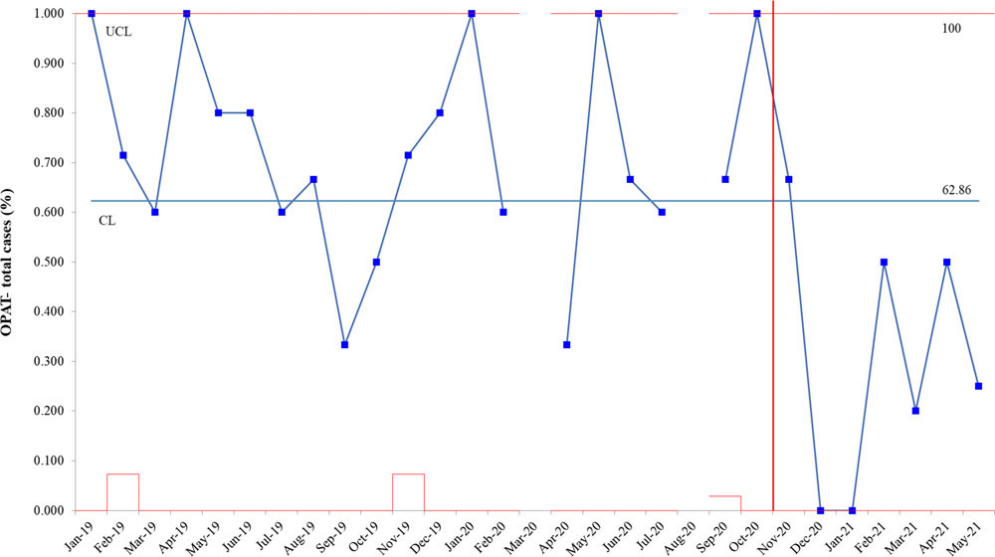
Monthly proportion of patients who received intravenous antibiotics.

Our protocol had 2 major components. A reminder e-mail was sent to the ID staff clinician on service every week regarding the quality improvement initiative with the consensus table. Real-time, case-based discussions were then held between the project leader (H.S.) and the ID provider on service.

All patients with BJIs were identified through the antimicrobial stewardship team’s prospective audit and feedback process, which is conducted daily on weekdays. It was complemented by the inpatient ID consultation list over the weekend. We limited the case discussion (1) to stable patients with BJI who can take oral antibiotics and (2) to patients for whom there were available oral options based on positive microbiology data (ie, either blood culture or local tissue culture). We did not perform a case discussion for patients with conditions for which OPAT is usually preferred (eg, *S. aureus* bacteremia, a large undrained abscess, an epidural abscess, meningitis, endovascular infection, or negative culture results). These patients were still included in our outcome analysis.

### Measures

Patient data were collected through the computerized patient record system (CPRS). We collected age; comorbidities, which were aggregated as Charlson comorbidity index (CCI); the ID provider who made a final antibiotic recommendation; the type of recommended antibiotics (IV or oral); culture results; LOS after final ID recommendation; disposition at discharge (home or post-acute care facility); surgical treatment; recurrence; and death. Our implementation period was November 1, 2020, to May 31, 2021. As a preimplementation period, we obtained data for patients with a BJI from January 1, 2019, to October 31, 2020. If a patient had >1 BJI over the study period, only the first episode was included. Our primary outcome was the proportion of patients treated with OPAT. We considered the following secondary outcomes: LOS after final ID recommendation, total LOS, the proportion of patients discharged to a facility, and recurrence or death within 6 months from the day of the ID service’s final recommendation. Recurrence was defined as an escalation of antibiotic therapy in the setting of worsening infection or reinitiation of antibiotics after completion of therapy for reasons other than perioperational antibiotics for planned surgery. We did not consider planned surgeries as treatment failure because inpatient surgical debridement for peripheral osteomyelitis tended to be deferred until after hospital discharge due to the lack of an inpatient podiatry service at ICVAHCS.

### Analysis

A comparison between the preimplementation period and implementation periods was performed using the Student *t* test for continuous variables and the χ^2^ test or the Fisher exact test for categorical variables, as appropriate. The monthly number and proportion of patients discharged on OPAT among all BJI patients were displayed with statistical process control (SPC) charts. Statistical analyses were conducted using R version 3.5.0 software (R Foundation for Statistical Computing, Vienna, Austria) and QI Macros Statistical Software (Denver, CO). This study was reviewed by the University of Iowa/ ICVAHCS Institutional Review Board and was determined to be a quality improvement initiative. A waiver of informed consent was granted.

## Results

In total, 77 patients during the preimplementation period and 22 patients during the implementation period were identified to have a BJI (Table 1). We detected no significant differences in age, CCI, diagnosis, or culture results between the 2 periods. In both periods combined, peripheral osteomyelitis accounted for 62.6% of all cases, including 46 (58.4%) in the preimplementation period and 16 (72.7%) in the implementation period. Furthermore, 70.1% of patients during the preimplementation period received OPAT, whereas only 31.8% of patients received OPAT during the implementation period (*P* = .003). The median LOS after the final ID recommendation was significantly shorter during the implementation period (median 3 days vs 1 day; *P* < .001), whereas the difference was not significant for total LOS (median 7 days vs 6 days; *P* = .06). The proportion of patients discharged home was higher in the implementation period, although the difference was not statistically significant (66.2% vs 86.4%; *P* = .07). We detected no significant difference in the 6-month rate of recurrence (24.7% vs 31.8%; *P* = .46) or mortality (9.1% vs 9.1%; *P* = 1.00). The monthly proportion of patients who had OPAT among all eligible patients with BJI is shown in Figure 1. Compared to the preimplementation period, there was a significant decrease in the proportion of patients who were started on OPAT during the implementation period.

**Table 1.** Comparison of Patient’s Characteristic Between the Preimplementation and Implementation Periods

Variable	Before Implementation (1/2019–10/2020)	During Implementation (11/2020–5/2021)	*P* Value
Total	77	22	
Median age, y (IQR)	70.0 (64.0–74.0)	68.0 (60.5–73.8)	.50
Sex, male, no. (%)	77 (100)	20 (90.9)	.05
Median Charlson comorbidity index (IQR)	4 (2–5)	2.5 (2–4)	.43
**Diagnosis, no. (%)**			.31
Native vertebral osteomyelitis	9 (11.7)	1 (4.5)	
Peripheral osteomyelitis	46 (58.4)	16 (72.7)	
Septic arthritis (native joint)	14 (18.1)	3 (13.6)	
Prosthetic joint infection	9 (11.7)	1 (4.5)	
Hardware-related infection	0 (0)	1 (4.5)	
**Local culture, no. (%)**			
*Staphylococcus aureus*	22 (28.6)	9 (40.9)	.40
Gram-negative rods	24 (31.2)	5 (22.7)	.62
Others	30 (39.0)	8 (36.4)	1.00
Negative or not taken	29 (36.4)	8 (36.4)	1.00
**Blood culture, no. (%)**			
*Staphylococcus aureus*	8 (10.4)	2 (9.1)	1.00
Gram-negative rods	4 (5.2)	2 (9.1)	.61
Others	6 (7.8)	1 (4.5)	1.00
Negative or not taken	60 (77.9)	17 (77.3)	1.00
**Intravenous or oral antibiotics, no. (%)**			.003
Intravenous	54 (70.1)	7 (31.8)	
Oral	23 (29.9)	15 (68.2)	
Surgery during treatment	52 (67.5)	13 (59.1)	.46
Median length of stay after final ID recommendation, d (IQR)	3 (2–5)	1 (1–2)	<.001
Total length of stay, d (IQR)	7 (5–12.0)	6 (4.3–8.8)	.06
**Disposition, no. (%)**			.07
Facility	26 (33.8)	3 (13.6)	
Home	51 (66.2)	19 (86.4)	
**Recurrence within 6 mo, no. (%)**			.46
Yes	19 (24.7)	7 (31.8)	
**Death within 6 mo, no. (%)**			1.00
Yes	7 (9.1)	2 (9.1)	

Among the 38 patients who were treated with oral antibiotics, the most commonly selected antibiotic was amoxicillin–clavulanate (20 patients) followed by fluoroquinolones (16 patients), doxycycline (13 patients), metronidazole (13 patients), linezolid (2 patients) and trimethoprim–sulfamethoxazole (2 patients). Also, >2 oral antibiotics were used in 16 patients. In patients for whom fluoroquinolones were used for treatment, they were used to treat GNR in 10 of 16 cases. In the 6 remaining cases, fluoroquinolones were used as an empiric therapy because no organism was identified.

During the implementation period, a case discussion was held for 8 patients (36.4%). After these discussions, 3 cases were treated with OPAT and 5 cases were treated with oral therapy. The following reasons were given for not holding a case discussion: no culture result to guide therapy (9 patients), final recommendation from the ID service had already been made (3 patients), and *S. aureus* bacteremia (2 patients).

In a sensitivity analysis limited to patients with peripheral osteomyelitis, the proportion of patients who had OPAT was 57.8% in the preimplementation period and 25.0% in the implementation period, respectively (*P* = .05). The median LOS was still significantly shorter in the implementation period: 2 days versus 1 day (*P* = .01). Surgical debridement was performed during treatment in 37 (60.7%) cases, and recurrence rates did not differ between the 2 periods (Supplementary Table 1 online).

The comparison of patients who received OPAT and with those who received oral antibiotics is shown in Table 2. A significantly higher proportion of patients who received oral therapy had peripheral osteomyelitis: 49.2% in the OPAT group versus 81.6% in oral antibiotics group (*P* = .003). The 6-month rate of recurrence was higher in the oral antibiotic group although the difference was not statistically significant: 21.3% in the OPAT group versus 34.2% in the oral antibiotics group (*P =* .24). Details of patients who had recurrence or death within 6 months are listed in Table 3.

**Table 2.** Comparison of Patient’s Characteristics Between Those Who Received Outpatient Parenteral Antimicrobial Therapy (OPAT) and Those Who Received Oral Antibiotics

Variable	OPAT	Oral Antibiotics	*P* Value
Total	61	38	
Median age, y (IQR)	70.0 (63.0–73.0)	70.0 (66.0–74.0)	.36
Sex, male, no. (%)	59 (96.7)	38 (100)	.52
Median Charlson comorbidity index (IQR)	3 (2–5)	4 (2.3–5)	.22
**Diagnosis, no. (%)**			.003
Peripheral osteomyelitis	30 (49.2)	31 (81.6)	
Other diagnoses	31 (50.8)	7 (18.4)	
Culture positive for *Staphylococcus aureus*, no. (%)	22 (36.1)	13 (34.2)	1.00
Culture positive for gram-negative rods, no. (%)	16 (26.2)	15 (39.5)	.25
Culture-negative or not taken, no. (%)	11 (18.0)	3 (7.9)	.24
Surgery during treatment, no. (%)	44 (72.1)	21 (55.3)	.13
Recurrence within 6 mo, no. (%)			.24
Yes	13 (21.3)	13 (34.2)	
Death within 6 mo, no. (%)			.73
Yes	5 (8.2)	4 (10.5)	

**Table 3.** Description of 31 Patients Who Experienced Recurrence or Death Within 6 Months

Age (Years) and Sex	Diagnosis	Culture Results	Antibiotics Recommended by ID Physician	Surgery During Treatment	Treatment Category	Pre or Post	Outcome	Description
78 M	Peripheral osteomyelitis	Tissue: *Streptococcus anginosus*, *Citrobacter koseri* and anaerobes	Ceftriaxone +metronidazole	Yes	OPAT	Pre	Recurrence	Patient with foot osteomyelitis was treated with partial toe amputation and 8 weeks of IV to oral antibiotics. Two months later, he presented with foot cellulitis and was later diagnosed with osteomyelitis.
57 M	Peripheral osteomyelitis	Tissue: *Staphylococcus epidermidis, Corynebacterium striatum*	Vancomycin	Yes	OPAT	Pre	Recurrence	Patient with foot osteomyelitis was treated with toe amputation and 8 weeks of IV antibiotics until second amputation. One week after second surgery, he was diagnosed with cellulitis and later with osteomyelitis.
66 M	Peripheral osteomyelitis	Tissue: *Streptococcus agalactiae, Enterococcus faecalis*	Piperacillin– tazobactam	Yes	OPAT	Pre	Recurrence	Patient with foot osteomyelitis was treated with IV therapy for 5 weeks until toe amputation. One month after the surgery, he was diagnosed with recurrence of osteomyelitis.
60 M	Peripheral osteomyelitis	Tissue: MSSA. *Streptococcus anginosus, Proteus mirabilis* and anaerobes	Ertapenem	Yes	OPAT	Pre	Recurrence	Patient with foot osteomyelitis was treated with IV to oral therapy for 12 weeks until toe amputation. One month after the surgery, he was diagnosed with recurrence of osteomyelitis.
66 M	Peripheral osteomyelitis	Tissue: *Streptococcus anginosus, Proteus mirabilis, Morganella morganii*	Ceftriaxone	Yes	OPAT	Pre	Death	Patient with foot osteomyelitis was treated with incision and drainage and IV antibiotics. He died while on IV therapy. No sign of recurrent infection.
71 M	Peripheral osteomyelitis	Tissue: MRSA, *Pseudomonas aeruginosa*, *Proteus* spp., *Enterococcus faecalis, Corynebacterium* spp.	Vancomycin +cefepime +metronidazole	Yes	OPAT	Pre	Recurrence and death	Patient with stump infection after BKA was treated with revision of BKA and IV antibiotics. While on IV therapy, his condition and he needed AKA. Patient died due to renal failure during hospitalization after AKA.
64 M	Peripheral osteomyelitis	Tissue: MSSA, *Enterobacter cloacae*	Ertapenem	No	OPAT	Pre	Recurrence	Patient with foot osteomyelitis was treated with IV to oral therapy for 10 weeks without surgery. Two months after antibiotic therapy, he was admitted with concern for worsening osteomyelitis.
62 M	Peripheral osteomyelitis	Blood: MRSA	Daptomycin	No	OPAT	Pre	Death	Patient with hand osteomyelitis and MRSA bacteremia/endocarditis died due to generalized decline in status while on IV therapy. No recurrence of bacteremia was recorded.
67 M	Peripheral osteomyelitis	Blood: MSSA	Cefazolin +metronidazole	No	OPAT	Pre	Recurrence	Patient with foot osteomyelitis and MSSA bacteremia was treated with 12 weeks of IV to oral antibiotics until toe amputation. One week after amputation, patient developed cellulitis and later osteomyelitis. Patient was restarted on IV antibiotics.
70 M	Peripheral osteomyelitis	Tissue: *Staphylococcus lugdunensis*	Ceftriaxone +metronidazole	Yes	OPAT	Pre	Recurrence	Patient with foot osteomyelitis treated with toe amputation and 8 weeks of IV to oral antibiotics. He experienced toe gangrene 2 months after completion of therapy.
72 M	Peripheral osteomyelitis	Tissue: *Enterobacter cloacae, Morganella morganii*	Ciprofloxacin	Yes	Oral	Pre	Recurrence	Patient with foot osteomyelitis was treated with toe amputation with 6 weeks of oral therapy. After 1 month of antibiotic therapy, he presented with a flare of osteomyelitis.
70M	Peripheral osteomyelitis	Blood: *Streptococcus mitis/oralis*	Amoxicillin/ clavulanate	Yes	Oral	Pre	Recurrence	Patient with foot osteomyelitis was treated with incision and drainage with 6 weeks of oral therapy. After 2 months of antibiotic therapy, he presented with a flare of osteomyelitis.
67 M	Peripheral osteomyelitis	Negative	Amoxicillin/ clavulanate +doxycycline	No	Oral	Pre	Recurrence	Patient with foot osteomyelitis was treated with 9 weeks of oral therapy but had worsening of wound while on therapy, which required transition to IV therapy.
71 M	Peripheral osteomyelitis	Tissue: MSSA	Doxycycline	No	Oral	Pre	Recurrence	Patient with foot osteomyelitis and MSSA bacteremia was treated with 2 weeks of inpatient IV therapy and transitioned to 2 weeks of oral therapy. Four months later he had a flare of osteomyelitis and required BKA.
74 M	Peripheral osteomyelitis	Tissue: MSSA	Doxycycline	No	Oral	Pre	Recurrence and death	Patient with foot osteomyelitis by MSSA and was treated with 6 weeks of oral therapy without surgery. After 1 month of therapy, he developed MSSA bacteremia/endocarditis and died during hospitalization.
70 M	Peripheral osteomyelitis	Tissue: MSSA, *Serratia marcescens*	Moxifloxacin	Yes	Oral	Pre	Recurrence	Patient with foot osteomyelitis was treated with 6 weeks of oral therapy without surgery. After 2.5 month of therapy, he developed a flare of osteomyelitis.
76M	Peripheral osteomyelitis	Tissue*: Pseudomonas aeruginosa, Klebsiella oxytoca, Enterococcus faecalis*	Amoxicillin/ clavulanate +ciprofloxacin	Yes	Oral	Pre	Recurrence	Patient with foot osteomyelitis was treated with toe amputation with 6 weeks of oral therapy. After 2 months of antibiotic therapy, he presented with a flare of osteomyelitis.
70M	Peripheral osteomyelitis	Tissue: *Klebsiella oxytoca, Providencia stuartii, Alcaligenes faecalis*	Ciprofloxacin +metronidazole	No	Oral	Pre	Death	Patient with foot osteomyelitis was treated with 6 weeks of oral therapy without surgery. Patient died while on treatment. There was no sign of worsening infection.
54 F	Peripheral osteomyelitis	Blood: *Morganella morganii*, Tissue: *Morganella morganii*, MSSA, *Enterococcus faecalis*	Ertapenem	No	OPAT	Post	Recurrence	Patient with ankle osteomyelitis and *Morganella* bacteremia was treated with 8 weeks of IV to oral therapy. After 1 month of therapy, she presented again with a flare of osteomyelitis and bacteremia.
70 M	Peripheral osteomyelitis	Blood: MSSA	Linezolid	No	Oral	Post	Recurrence	Patient with foot osteomyelitis with MSSA bacteremia treated with 6 weeks of oral linezolid without surgery. Three weeks after completion, he was suspected to have toe cellulitis and was treated with 2 weeks of oral antibiotics.
64 M	Peripheral osteomyelitis	Tissue: MSSA, *Corynebacterium striatum, Finegoldia magna*	Amoxicillin/ clavulanate	No	Oral	Post	Recurrence	Patient with foot osteomyelitis was treated with 5 weeks of oral therapy without surgery. He experienced a flare of osteomyelitis 2 months later.
82 M	Peripheral osteomyelitis	Blood: *Proteus mirabilis*, Tissue: *Proteus mirabilis*, *Klebsiella pneumoniae, Enterococcus faecalis*	Amoxicillin/ clavulanate +ciprofloxacin	No	Oral	Post	Death	Patient with foot osteomyelitis was treated with 4 weeks of oral therapy without surgery. He died 3 months later from an unknown cause.
69 M	Peripheral osteomyelitis	Negative	Ciprofloxacin +doxycycline +metronidazole	Yes	Oral	Post	Recurrence	Patient with foot osteomyelitis was treated with 6 weeks of oral therapy with toe amputation. He had a flare of osteomyelitis 1.5 months after completion, which required further amputation.
70M	Peripheral osteomyelitis	Tissue: MSSA, *Pseudomonas aeruginosa, Enterococcus faecalis*	Amoxicillin/ clavulanate +ciprofloxacin	Yes	Oral	Post	Recurrence and death	Patient with foot osteomyelitis was treated with 4 weeks of oral therapy with toe amputation. Patient experienced relapse of osteomyelitis 1 month later. Patient died 1 month after relapse.
66 M	Peripheral osteomyelitis	Negative	Amoxicillin/ clavulanate +doxycycline	No	Oral	Post	Recurrence	Patient with foot osteomyelitis was treated with 6 weeks of oral therapy without surgery. Two months later he experienced a flare of osteomyelitis.
85 M	Peripheral osteomyelitis	Negative	Moxifloxacin +doxycycline	No	Oral	Post	Recurrence	Patient with foot osteomyelitis was treated with 6 weeks of oral therapy without surgery. Two weeks after completion, patient had amputation. There was some possible infection was present, and he received a course of antibiotics for the remaining soft-tissue infection.
53 M	Prosthetic joint infection	Negative	Ceftaroline	Yes	OPAT	Pre	Recurrence	Patient with recurrent prosthetic joint infection was treated with removal of prothesis and spacer placement with 6 weeks of IV antibiotics. Two months after antibiotic treatment, he was diagnosed with another PJI.
68 M	Prosthetic joint infection	Negative	Cefazolin	Yes	OPAT	Pre	Recurrence	Patient with PJI was treated with IV antibiotics which was stopped at 3 weeks, then he had a recurrence 1 week later.
65 M	Prosthetic joint infection	Blood and tissue: MSSA	Cefazolin +rifampin	Yes	OPAT	Pre	Recurrence	Patient with prosthetic joint infection and MSSA bacteremia was treated with incision and drainage, poly-exchange and 6 weeks of IV antibiotics. Patient experienced another prosthetic joint infection 1 month after IV therapy and while on suppressive oral antibiotic.
76 M	Vertebral osteomyelitis	Negative	Vancomycin + ceftriaxone	No	OPAT	Pre	Death	Patient with thoracic spine osteomyelitis died while on planned 6 weeks of IV therapy. Reason for death was not clear.
72 M	Septic arthritis	Blood and tissue: MRSA	Daptomycin	Yes	OPAT	Pre	Recurrence and death	Patient with septic arthritis and MRSA bacteremia was treated with 8 weeks of IV therapy. He had recurrence of MRSA bacteremia after 1 week of completion of therapy and died.

Note. M, male; F, female; ID, infectious diseases; MSSA, methicillin-susceptible *Staphylococcus aureus*; MRSA, methicillin-resistant *Staphylococcus aureus*; OPAT, outpatient parenteral antimicrobial therapy; IV, intravenous; PJI, prosthetic joint infection; BKA, below-knee amputation; AKA, above-knee amputation.

## Discussion

Infectious disease healthcare providers used significantly less OPAT for BJIs after implementation of our protocol, which included weekly e-mail reminders and case-based discussions. The LOS after the final ID recommendation was significantly shorter during the implementation period. Clinical outcomes, defined as recurrence or death during the 6 months after final ID recommendations, were not significantly different between the preimplementation period and the implementation period.

Increased use of oral antibiotics for BJI in this study may imply that ID providers are changing their practice and moving away from the traditional dogma “IV therapy is required for BJI” and accepting oral antibiotics for BJI. Although we saw a rapid decrease in OPAT use after application of our protocol, we do not think our protocol changed ID provider practice solely by itself. The protocol was implemented nearly 2 years after the OVIVA trial was published, and providers may have already been starting to change their practice. Another important factor that could have affected the shift to oral therapy was the coronavirus disease 2019 (COVID-19) pandemic. The COVID-19 pandemic could have made ID providers more likely to choose oral antibiotics to avoid physical, in-person visits or nursing home placement to administer OPAT. Nevertheless, we believe that the process of developing the protocol and its implementation augmented ID providers’ acceptance for oral antibiotics through presenting evidence for the utility of oral antibiotics. Our experience suggests that developing hospital-specific guidance for BJIs, which draws input from multiple stakeholders, may be an effective implementation strategy for incorporating the OVIVA findings into routine medical care.^19^

The most common oral antibiotics used for treatment of BJI in our study was amoxicillin–clavulanate. Because most of our cases were peripheral osteomyelitis (eg, diabetic foot infections, which are frequently polymicrobial), it is reasonable that amoxicillin–clavulanate, which covers gram-positive pathogens, gram-negatives pathogens, and anaerobes, was frequently used. The second most used antibiotic was fluoroquinolones. Interestingly, this class was used mainly to treat gram-negative infection or a part of empiric therapy but did not seem to be used for *Staphylococcus* infection. This finding is somewhat contrary to the OVIVA study in which fluoroquinolones were used for >40% of cases, including BJIs caused by *S. aureus*.^10^ The difference could be at least partially explained by the reluctance of ID providers to use fluoroquinolones for *S. aureus* due to the US Food and Drug Administration (FDA) warnings about the side effects of fluoroquinolones,^20,21^ the unnecessarily broad spectrum these agents provide, and the increasing resistance of *S. aureus* to fluoroquinolones in the United States.^22^ In addition, a previous study in the United States reported far more unintended drug discontinuation with fluoroquinolone-based regimens for prosthetic joint infections compared to non–fluoroquinolone-based regimens. It is possible that ID providers are not comfortable in selecting fluoroquinolone as a drug of choice for *S. aureus* BJI in the United States.^23^

Our 6-month recurrence rates were 21.3% in patients treated with OPAT and 34.2% in patients treated with oral antibiotics. These recurrence rates were higher than those reported in the OVIVA trial, which reported 1-year recurrence rates of 14.6% in OPAT group and 13.2% in the oral antibiotic group.^10^ The difference can be at least partially explained by the different types of BJI between the 2 cohorts and the lower rate of timely surgical treatment in our study compared to the OVIVA trial. Although most of our patients had peripheral osteomyelitis, ∼60% of patients in the OVIVA trial had prosthetic joint infections or orthopedic device–related infections, all of whom underwent some form of debridement. In addition, only ∼60% of patients with peripheral osteomyelitis in our cohort received timely surgical treatment; this was much lower than that of the OVIVA trial, which reported that 85.6% of patients with chronic osteomyelitis underwent debridement.^10^ It is possible that peripheral osteomyelitis carries a higher recurrence rate without timely surgical treatment. Based on these findings, the ID providers at the ICVAHCS are engaging our local orthopedic and podiatry services to discuss how patients with peripheral osteomyelitis, namely due to diabetic foot infections, can receive more timely surgical interventions.

This study had several limitations. It was a single-center study within the VA healthcare system, and the number of patients included in this quality improvement initiative was relatively small. We were not able to continue the implementation longer because of the lack of available resources. Therefore, it is possible that we could not detect a true difference in clinical outcomes due to type II error. In fact, the 6-month rate of recurrence was higher in the oral antibiotic group compared to the OPAT group, although the difference was not statistically significant. The difference might have been confounded by the fact that oral antibiotics were used more often for peripheral osteomyelitis and that many of these patients did not have timely surgical debridement. We were unable to perform a more robust analysis (eg, an interrupted time-series analysis) due to the small number of patients. Our local practice might have affected the results. For example, because there is not an inpatient podiatry service at the ICVAHCS, many patients with diabetic foot osteomyelitis could not have timely surgical debridement while hospitalized. This factor might have affected some decisions by ID provider to administer IV therapy because source control was not complete, and this could have increased the chance for recurrence in those patients with peripheral osteomyelitis. We used recurrence or death within 6 months from the ID service’s final recommendation as the definition of clinical failure. This time frame was shorter than that of some previous studies that followed patient outcomes for 1 year. BJIs are sometimes caused by indolent organisms and recur after months of antibiotic therapy. Furthermore, we could not detect a recurrence if the patient received care outside the VA system. For those reasons, we may have underestimated the occurrence of clinical failure. We were unable to assess whether unmeasured factors, such as the COVID-19 pandemic, affected the practice change we observed. Lastly, we did not collect other relevant information such as adverse reactions associated with IV and oral antibiotics, cost associated with antibiotic treatment, or patient satisfaction.

In conclusion, more patients admitted with BJIs were treated with oral antibiotics during the implementation phase of our quality improvement initiative. The presence of an antibiotic stewardship team provided a structure to identify and encourage oral treatment for eligible patients. Although the use of more oral antibiotics led to shorter LOS and likely led to more patients discharged home, the overall recurrence rate on oral therapy was higher than we anticipated, which may reflect the high proportion of cases with peripheral osteomyelitis and the inconsistent performance of surgical debridement. BJI is heterogenous, and in some situations oral therapy can be safely used as an alternative to IV therapy, but in other situations IV therapy is still better. Larger studies will be needed to validate our findings regarding clinical outcomes and to further investigate the best situations in which oral antibiotics can be used for BJI.

## Supplementary material

For supplementary material accompanying this paper visit https://doi.org/10.1017/ash.2021.250. click here to view supplementary material
