# Supplementary material for: Implementation of a consensus protocol for antibiotic use for bone and joint infection to reduce unnecessary outpatient parenteral antimicrobial therapy: A quality improvement initiative
Source: Antimicrob Steward Healthc Epidemiol. 2022 Jan 12;2(1):e6. doi: 10.1017/ash.2021.250 (PMC9614991; doi:10.1017/ash.2021.250)
Supplement: Supplementary file 1 [file S2732494X21002503sup001.docx]

Supplemental Table 1. Comparison of patient’s characteristic between pre-implementation and implementation periods, limited to patients with peripheral osteomyelitis

|  | Pre-implementation (1/2019-10/2020) | During implementation (11/2020-5/2021) | p-value |
| --- | --- | --- | --- |
| Total number | 45 | 16 |  |
| Median age (IQR) | 70.0 (66.0-73.0) | 66.5 (61.5-71.0) | 0.48 |
| Male (%) | 45 (100) | 14 (87.5) | 0.07 |
| Median Charlson Comorbidity Index (IQR) | 4 (3-5) | 2.5 (2-4.3) | 0.02 |
| Local culture (%) | | |  |
| *Staphylococcus aureus* | 17 (37.8) | 5 (31.3) | 0.87 |
| Gram-negative rods | 19 (42.2) | 5 (31.3) | 0.64 |
| Others | 23 (51.1) | 7 (43.8) | 0.83 |
| Negative or not taken | 11 (24.4) | 7 (43.8) | 0.26 |
| Blood culture (%) |  |  |  |
| *Staphylococcus aureus* | 5 (11.1) | 1 (6.3) | 1.00 |
| Gram-negative rods | 1 (2.2) | 2 (12.5) | 0.17 |
| Others | 3 (6.7) | 0 (0) | 0.56 |
| Negative or not taken | 36 (80.0) | 13 (81.3) | 1.00 |
| Intravenous or oral antibiotics (%) | | | 0.05 |
| Intravenous | 26 (57.8) | 4 (25.0) |  |
| Oral | 19 (42.2) | 12 (75.0) |  |
| Surgery during treatment (%) | 30 (66.7) | 7 (43.8) | 0.14 |
| Median length of stay after final ID recommendation, days (IQR) | 2 (1-4) | 1 (1-2) | 0.01 |
| Total length of stay, days (IQR) | 7.5 (5-14) | 6 (4.8-8.5) | 0.11 |
| Disposition (%) | | | 0.31 |
| Facility | 13 (28.9) | 2 (12.5) |  |
| Home | 32 (71.1) | 14 (87.5) |  |
| Recurrence within 6 months (%) | | | 0.55 |
| Yes | 15 (33.3) | 7 (43.8) |  |
| Death within 6 months (%) | | | 1.00 |
| Yes | 5 (11.1) | 2 (12.5) |  |

Supplemental Figure 1. Consensus table of oral antibiotics for bone and joint infections

**Antibiotic treatment options for bone and joint infections**

| Organism* | MSSA, MSCoNS | MRSA, MRCoNS | Streptococcus | Enterococcus | Enteric GNR | Pseudomonas | Organism unknown |
| --- | --- | --- | --- | --- | --- | --- | --- |
| First-line  oral options | Linezolid  TMP-SMX  Doxycycline  Rifampin can be added for synergy^b^ | Linezolid  TMP-SMX  Doxycycline  Rifampin can be added for synergy^b^ | Linezolid  Clindamycin  Amoxicillin | Linezolid  Amoxicillin | Ciprofloxacin  TMP-SMX | Ciprofloxacin |  |
| Second-line oral options | Cephalexin  Dicloxacillin  Amox/Clav  Clindamycin  Levofloxacin^a^  +Rifampin | Clindamycin  Levofloxacin^a^ + Rifampin | Penicillin  Cephalexin  Levofloxacin |  |  |  | Levofloxacin + rifampin or Levofloxacin +clindamycin^c^ |

Footnotes

* Agent should ultimately be chosen based on various factors including susceptibility results, patient allergy profile, etc. Organisms was identified by either blood culture, bone biopsy or deep tissue culture

**First-line oral options** – either recommended in guidelines^1-3^, known to achieve high concentration in bone^4,5^, or showed comparative treatment effect compared to IV therapy^6,7^. Can be used as a monotherapy (except rifampin) or combination with other agents

**Second-line oral options** – theoretically used or reported in small studies^8^. Consider use in a combination therapy.

A – some studies use ciprofloxacin instead of levofloxacin as a combination therapy

B – some study argues decreased concentration of linezolid, clindamycin or TMP-SMX when combined with rifampin

C – European studies frequently use these regimens for empiric therapy, in contrast, Appropriateness in Korean study was about 70% (where MRSA more prevalent)

Development process - We asked how frequently they would feel comfortable using the antibiotics as a REDCap survey The frequency was scored from 5 (very frequently), 4(frequently), 3(sometimes), 2(rarely) and 1(almost never). We summarized the scores and took an average. If it was scored ≥ 3.5, then the antibiotic was changed to the first line (if not already on the 1^st^ line), 3-3.5 then stay at the same category, and 2-3 then changed to the second line (if not already on the 2^nd^ line). If the score was less than 2, the antibiotic was removed from the table.

**1.** Berbari EF, Kanj SS, Kowalski TJ, et al. 2015 Infectious Diseases Society of America (IDSA) Clinical Practice Guidelines for the Diagnosis and Treatment of Native Vertebral Osteomyelitis in Adults. *Clinical infectious diseases : an official publication of the Infectious Diseases Society of America* 2015;61:e26-46.

**2.** Osmon DR, Berbari EF, Berendt AR, et al. Diagnosis and management of prosthetic joint infection: clinical practice guidelines by the Infectious Diseases Society of America. *Clinical infectious diseases : an official publication of the Infectious Diseases Society of America* 2013;56:e1-e25.

**3.** Lipsky BA, Berendt AR, Cornia PB, et al. 2012 Infectious Diseases Society of America clinical practice guideline for the diagnosis and treatment of diabetic foot infections. *Clinical infectious diseases : an official publication of the Infectious Diseases Society of America* 2012;54:e132-173.

**4.** Spellberg B, Lipsky BA. Systemic antibiotic therapy for chronic osteomyelitis in adults. *Clinical infectious diseases : an official publication of the Infectious Diseases Society of America* 2012;54:393-407.

**5.** Senneville E, Robineau O. Treatment options for diabetic foot osteomyelitis. *Expert opinion on pharmacotherapy* 2017;18:759-765.

**6.** Li HK, Rombach I, Zambellas R, et al. Oral versus Intravenous Antibiotics for Bone and Joint Infection. *The New England journal of medicine* 2019;380:425-436.

**7.** Euba G, Murillo O, Fernandez-Sabe N, et al. Long-term follow-up trial of oral rifampin-cotrimoxazole combination versus intravenous cloxacillin in treatment of chronic staphylococcal osteomyelitis. *Antimicrobial agents and chemotherapy* 2009;53:2672-2676.

**8.** Oh WS, Moon C, Chung JW, et al. Antibiotic Treatment of Vertebral Osteomyelitis caused by Methicillin-Susceptible Staphylococcus aureus: A Focus on the Use of Oral beta-lactams. *Infection & chemotherapy* 2019;51:284-294.
